# Editorial: New challenges and perspectives in emotion regulation and processing

**DOI:** 10.3389/fnbeh.2022.1006627

**Published:** 2022-09-09

**Authors:** Wenhai Zhang, Gustavo Provensi, Antonio Maffei

**Affiliations:** ^1^College of Educational Science, Hengyang Normal University, Hengyang, China; ^2^Shanghai Key Laboratory of Mental Health and Psychological Crisis Intervention, School of Psychology and Cognitive Science, East China Normal University, Shanghai, China; ^3^Department of Neurosciences, Psychology, Drug Research and Child Health (NEUROFARBA), University of Florence, Florence, Italy; ^4^Department of Developmental Psychology and Socialization (DPSS) and Padova Neuroscience Center (PNC), University of Padova, Padova, Italy

**Keywords:** emotion regulation, emotional intelligence, emotional exhaustion, event-related potential (ERP), leadership

Emotion regulation and processing play a crucial role in maintaining mental health, promoting individual development, and fostering successful social relationships. Over the last decade, a growing number of theories and experimental paradigms in this field have been proposed, which brings some new challenges. With the development of neuroscience technologies, an increasing number of neuroimaging studies have revealed that dysfunctions within multiple brain regions precipitates affective disorders. Cross-disciplinary advances in psychological, clinical, and organizational management also provide an opportunity for further understanding the multilevel mechanisms of emotion regulation and processing.

To integrate these theories and models and further promote the development of this field, we proposed the Research Topic “*New challenges and perspectives in emotion regulation and processing*,” which provided a platform for collecting recent discoveries from developmental, neuroimaging, clinical, and organizational management research.

In the process model of emotion, emotion regulation, whether deliberate or automatic, involves changes at all levels of the emotion-generation process. As an unconscious, implicit, or impulsive process, automatic emotion regulation is defined as the changes in any aspect of an individual's emotional responses without conscious intent. Event-related potential has excellent temporal resolution to be suitable for characterizing the temporal processing of automatic regulation. In the cued-emotion Go/Nogo paradigm, Liu et al. selected 65 children aged 8–12 and found that emotional faces in Nogo trials evoked greater N2 and P3 amplitudes than neutral faces, reflecting cognitive conflict and automatic response inhibition to emotions, respectively. No significant difference in N2 and P3 amplitudes was found in Go trials, indicating that children aged 8–12 showed similar automatic top-down control and similar motivated attention, respectively. They also revealed that Nogo-P3 induced by neutral pictures was positively correlated with temperamental subcomponents (e.g., negative affect and social anxiety). These findings suggest that 8- to 12-year-old children automatically regulate emotions that are modulated by temperament.

In the predictive model of emotion, people use environmental cues and their previous experience to construct affective predictions (generation stage), then anticipate incoming stimuli and prepare action plans to deal with the expected situation (implementation stage), and finally adjust future predictions (updating stage) (Seth and Friston, 2016). Cristaldi et al. used an S1-S2 paradigm with emotional faces and pictures as S1s and S2s and manipulated contextual uncertainty across three blocks with 100, 75, or 50% S1-S2 affective congruency. They found that no emotional regulation (ER) strategy affected prediction generation. During implementation, expressive suppression predicted higher activity in the left supplementary motor area at 1,500–2,000 ms poststimulus and a smaller amplitude of contingent negative variation at 2,000–2,500 ms in the 75% block. During updating, cognitive reappraisal predicted larger P2, late positive potential, and right orbitofrontal cortex activity in the 75% block. These results suggest that both ER strategies interact with the levels of contextual uncertainty to differentially modulate event-related potentials (ERPs) and source activity, supporting the efficient updating of affective predictive models only in the context in which model updating actually occurs.

In the dual process model of depression (Beevers, 2005), early depressive vulnerability increases the individual risk for a depressive episode, while the inhibition mechanism fails during full-blown clinical depression. Facial expressions simultaneously transmit and evoke emotion, and people at risk of developing clinical depression exhibit attentional biases for emotional faces. However, it is unclear whether such effects occur at an early, automatic or at a late, deliberate processing stage of emotional processing. Jaspers-Fayer et al. found that abnormal changes in face processing in dysphoric individuals can occur at early (N170) stages of face perception, while the intermediate (EPN) and late (LPP) stages of emotion face processing appear unaffected by dysphoria. These clinical results support the idea that dysphoria may influence the stage of automatic emotional appraisal.

Organizational studies are investigating how to improve employee positive behavior. Emotional intelligence is regarded as the ability to use and regulate emotions and to evaluate emotions of others and one's own. Such ability can change over time and can be cultivated through education and training. Moreover, high levels of emotional intelligence mean that employees possess more emotional resources and emotional capacity to cope with negative behaviors (e.g., job burnout) and exhibit positive behaviors (e.g., job performance). Based on social learning theory, Yen proposed a theoretic model in which leadership influences emotional intelligence development, which in turn influences job performance development and job burnout development. This model would guide enterprises on how to build up employees' emotional intelligence through leadership mechanisms to fulfill organizational performance.

Emotional exhaustion means a feeling of exhaustion for emotional resources and influences many economic factors, e.g., negative work behavior and turnover intention. However, few studies have applied the emotional regulation perspective to explore intervention strategies for emotional exhaustion. Based on work engagement theory, Hu et al. proposed an emotion regulation model in which emotional leadership can improve emotional engagement and then decrease emotional exhaustion; emotional resources can moderate the relationship between emotional engagement and emotional exhaustion. Thus, contemporary enterprises should merge emotional leadership into education training projects to increase intervention in the emotional exhaustion of employees.

## Author contributions

WZ wrote the editorial, which was edited by GP and AM. All authors contributed to the article and approved the submitted version.

## Funding

This topic was funded by Hunan Natural Science Foundation of China (2022JJ30099), the Research Project of Shanghai and Technology Commission (20dz2260300), and the Fundamental Research Funds for the Central Universities.

## Conflict of interest

The authors declare that the research was conducted in the absence of any commercial or financial relationships that could be construed as a potential conflict of interest.

## Publisher's note

All claims expressed in this article are solely those of the authors and do not necessarily represent those of their affiliated organizations, or those of the publisher, the editors and the reviewers. Any product that may be evaluated in this article, or claim that may be made by its manufacturer, is not guaranteed or endorsed by the publisher.
